# Circ_0072088 Promotes Proliferation, Migration, and Invasion of Esophageal Squamous Cell Cancer by Absorbing miR-377

**DOI:** 10.1155/2020/8967126

**Published:** 2020-09-29

**Authors:** Na Fang, Yijun Shi, Yu Fan, Tao Long, Yongqian Shu, Jianwei Zhou

**Affiliations:** ^1^Department of Oncology, The First Affiliated Hospital of Nanjing Medical University, Nanjing 210029, China; ^2^Department of Molecular Cell Biology and Toxicology, Center for Global Health, School of Public Health, Nanjing Medical University, Nanjing 211166, China; ^3^Department of Thoracic and Cardiovascular Surgery, The Affiliated People's Hospital, Jiangsu University, Zhenjiang 212002, China; ^4^Institute of Molecular Biology & Translational Medicine, The Affiliated People's Hospital, Jiangsu University, Zhenjiang 212002, China; ^5^Jiangsu Key Lab of Cancer Biomarkers, Prevention and Treatment, Collaborative Innovation Centre For Cancer Medicine, Nanjing Medical University, Nanjing 211166, China

## Abstract

Circular RNA (circRNA) is an endogenous noncoding RNA. Accumulative investigations have confirmed that circRNAs play a vital role in carcinogenesis and tumor progression. Herein, we examined the expression and mechanism of circ_0072088 in esophageal squamous cell carcinoma (ESCC). As a result, circ_0072088 was significantly overexpressed in ESCC tissues and cells, which was closely associated with tumor size, invasion depth, clinical stage, and lymph node metastasis of esophageal cancer. Nuclear and cytoplasmic separation as well as FISH assays showed that circ_0072088 was mainly localized in the cytoplasm of ESCC cells. RNase R treatment assay revealed that circ_0072088 was steadier than linear ZFR mRNA. circ_0072088 promoted ESCC cell proliferation, migration and invasion *in vitro*, and cell proliferation *in vivo*. Mechanistically, circ_0072088 upregulated VEGF gene expression by acting as the sponge of miRNA-377. In conclusion, circ_0072088 might be used as a diagnostic biomarker and therapeutic target for ESCC.

## 1. Introduction

Esophageal cancer (EC) is one of the most common malignancies worldwide, ranking the seventh in terms of incidence and the sixth in terms of cancer-related death among the 10 most common malignancies globally [1]. EC is mainly divided into two major pathological categories: esophageal squamous cell carcinoma (ESCC) and esophageal adenocarcinoma (EAC), with ESCC accounting for as high as 90% [2]. Due to a lack of typical symptoms in the early stage in most EC patients, the diagnosis rate in the early stage is low, while the majority of EC patients are already in an advanced stage at diagnosis [3]. The 5-year survival rate of EC is only 29.7% in China [4]. Thus, it is an urgent need to investigate the pathogenesis of EC at the molecular level and to explore molecular markers as well as therapeutic targets for early diagnosis of EC.

Circular RNAs (circRNAs) are a novel type of noncoding RNAs existing in eukaryotes with abundant expression [5]. According to sources, circRNAs consist of exon circularization, intron circularization, and cocirculation of exons and introns, with exon circRNAs as the most common [6]. Unlike linear RNA, circRNAs have a covalently closed loop structure without 5′ to 3′ polarity, making it more resistant to RNase R, which also makes circRNA more stable than its linear counterparts in cells [7]. circRNAs are highly conserved among different species and specifically expressed in tissues [8]. A recent study has disclosed that the differential expressions of circRNAs affect the occurrence and progression of carcinomas [9–12], including the proliferation, migration, and invasion of EC [13, 14]. However, there are relatively limited studies concerning the effects of circRNA on ESCC overall.

Based on the differential expression of circRNA chip from 10 pairs of ESCC and matched adjacent tissues in our previous study [14], in this study, the top ten circRNAs with elevated expression were selected and further validated in cancer tissues and matched adjacent tissues of another 25 ESCC patients, revealing circ_0072088 with the most significant differential expression. circ_0072088 expression was elevated in ESCC tissues and cells and was positively correlated with malignant phenotypes both *in vitro* and *in vivo*. Therefore, circ_0072088 may importantly involve in the development of ESCC.

## 2. Materials and Methods

### 2.1. Collection of Specimens from ESCC Patients

Surgical samples were collected from 83 patients with pathologically diagnosed ESCC undergoing surgical resection in the Affiliated People's Hospital of Jiangsu University from January 2014 to December 2018. None of them underwent chemotherapy, radiotherapy, or other treatments before the surgical operation. Samples were collected within approximately 5 min after tumor resection and stored in liquid nitrogen. The study was gained approval from the Medical Ethics Committee of the Affiliated People's Hospital of Jiangsu University, and written informed consent was signed by all subjects.

### 2.2. Cell Culture

Five ESCC cells (TE-13, Kyse150, ECA109, Kyse450, and Kyse510) and normal esophageal epithelial cells HET-1A were commercially obtained from Cell Bank of the Chinese Academy of Sciences (Shanghai, China). All cell lines were maintained in RPMI1640 medium (GIBCO, USA) containing 10% fetal bovine serum (FBS) (GIBCO), 1% streptomycin, and 1% penicillin at 37°C in an incubator containing 5% CO_2_.

### 2.3. Quantitative Real-Time PCR (qRT-PCR)

Total RNA was extracted from cells and tissues by Trizol reagent (Invitrogen, Shanghai, China) and GeneJET RNA Purification Kit (Invitrogen), respectively. RT SuperMix Reverse Transcription Kit (Vazyme Biotech, Nanjing, China) was employed for reverse transcription from RNA into cDNA, followed by qRT-PCR using SYBR Premix Ex Taq II Kit (Vazyme Biotech). The relative expression was calculated by the 2^−ΔΔCT^ method, GAPDH was utilized to normalize the relative expression levels of circRNA and linear mRNA, and U6 was utilized to normalize the expression level of microRNA. The primer sequences were listed in Supplementary Table S1.

### 2.4. siRNA, miRNA, shRNA, and Plasmid Construction

The circ_0072088 overexpression plasmid, siRNAs targeting ZFR, and lentiviral shRNAs targeting circ_0072088 were synthesized by GenePharma (Shanghai, China). miRNA-377 mimic, miRNA-377 inhibitor, and the corresponding control sequences were also designed and synthesized by GenePharma. The sequences of sh-circ_0072088, si-ZFR, miR-377 mimic/NC, and miR-377 inhibitor/NC were shown in Supplementary Table S2.

### 2.5. Cell Proliferation Assay

Cell proliferation ability was examined by Cell Counting Kit-8 (CCK-8) as well as colony formation assay. In the CCK-8 assay, ESCC cells were inoculated into the 96-well plate (2000 cells/per well), and CCK-8 solution (Dojindo, Kumamoto, Japan) was added to each well at 24, 48, 72, and 96 h separately. Afterward, cells at each time point were incubated at 37°C for an additional 2 h, followed by measurement of absorbance at a wavelength at 450 nm. In the colony formation assay, 500 ESCC cells were inoculated in each well of the 6-well plates. After culture for 14 days, cells were then fixed, stained, photographed, and counted.

### 2.6. Transwell Assays

2 × 10^4^/100 *μ*l of ESCC cells in serum-free medium were added to the top Transwell chambers (8 *µ*m, pore size, Corning, NY, USA) with or without Matrigel (BD Biosciences, USA). In addition, the lower chambers were added with a medium containing 10% FBS. After 24 h, cells passing through the membrane were fixed, stained, photographed, and counted.

### 2.7. RNase R Treatment Assay

2 *µ*g RNA and 6-unit RNase R (Geneseed Biotech, Guangzhou, China) were mixed and incubated at 37°C for 20 min. qRT-PCR for determining the mRNA level of circ_0072088 and linear ZFR before and after RNase R treatment.

### 2.8. Nuclear and Cytoplasmic Separation Assay

PARIS kit (Invitrogen, Shanghai, China) was purchased to extract nuclear and cytoplasmic RNA in line with the standard protocol. Subsequently, the relative expression of circ_0072088 in the nucleus and cytoplasm was measured by qRT-PCR.

### 2.9. RNA Fluorescence In Situ Hybridization (FISH)

The cy3-labeled circ_0072088 probe was purchased from GenePharma. A FISH Kit was purchased (Ribobio, Guangzhou, China) and performed accordingly. Afterward, Zeiss AIM software along with a Zeiss LSM 700 confocal microscope system (Carl Zeiss Jena, Oberkochen, Germany) was utilized to capture confocal images of cells.

### 2.10. RIP Assay

EZMagna RIP kit (Merck, Darmstadt, Germany) was utilized. In brief, ESCC cells were lysed with RIP lysis buffer. The lysate was incubated with magnetic beads that had bounded with anti-Argonaute 2 (Ago2) or anti-IgG antibody at 4°C for 6 h. The magnetic beads-bound RNA was extracted, purified, and detected by qRT-PCR.

### 2.11. Dual-Luciferase Reporter Gene Assay

circ_0072088-wt or circ_0072088-mut was constructed into a dual-luciferase reporter plasmid (GP-miRGLO) (GenePharma). TE-13 cells were inoculated into 24-well plates (4 × 10^4^ cells/per well), followed by transfection after 24 h. Lipofectamine 2000 (Invitrogen) was used for cotransfection of dual-luciferase reporter plasmid and miR-377 mimics or miR-377 NC into TE-13 cells. Subsequently, luciferase activity was measured by a dual-luciferase reporter kit (Promega, WI, USA) after 48 h.

### 2.12. Western Blot Analysis

RIPA lysis buffer (Thermo Scientific, USA) was utilized for protein extraction from ESCC cells after transfection for 48 h, followed by protein quantification using a BCA kit (Beyotime Biotechnology, Nantong, China). Afterward, 20 *µ*g of protein sample was separated by electrophoresis, transferred to the PVDF membrane, and blocked in the blocking solution at 37°C on a shaker for 1 h. Afterward, membranes were reacted with primary antibody at 4°C overnight, reacted with proper secondary antibody at 37°C for 1 h, visualized by chemiluminescence (ECL) (YEASEN, Shanghai, China), and processed and analyzed by a gel imager. Tubulin was used as the internal control in the experiment. The antibodies used in Western blot were as follows: VEGF (1 : 1000, YEASEN), Tubulin (1 : 1000, Beyotime Biotechnology), and HRP-conjugated secondary antibody (1 : 1000, Beyotime Biotechnology). The assay was performed in triplicate.

### 2.13. Xenograft Mouse Model

The animal experiment procedure gained approval from the Animal Care and Use Committee of Jiangsu University. Six-week-old BALB/c nude mice were purchased from GemPharmatech (Nanjing, China) and housed in the SPF room in the Animal Experimental Center of Jiangsu University. Twelve mice were randomly divided into the sh-circ_0072088 group and the control group (*N* = 6 each). TE13 cells with stable expression of sh-circ_0072088 or sh-control were injected subcutaneously into the right axilla of mice (4 × 10^6^/each mouse). After seven days, the tumor volume of nude mice was measured once a week to plot the tumor growth curve. After 28 days, mice were anesthetized and sacrificed by cervical dislocation. The tumor mass was isolated and subjected to assays of immunohistochemistry (IHC).

### 2.14. Statistical Analysis

SPSS 21.0 software (IBM, Chicago, USA) was employed for statistical analysis. Experimental data were shown as mean ± standard deviation. The *t*-test was used for pairwise comparison, and Pearson's Chi-square test was utilized to analyze the correlation between circ_0072088 expression and clinical characteristics. A *P* < 0.05 indicated statistical significance.

## 3. Results

### 3.1. circ_0072088 Expression Was Increased in ESCC Tissues and Cells

**Figure 1:**
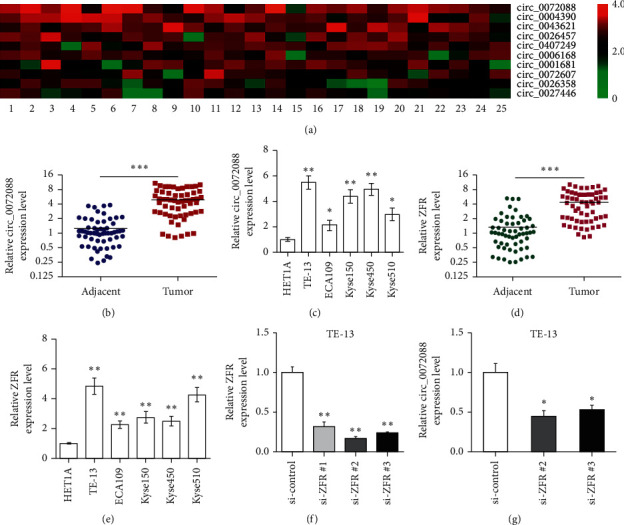
High expression of circ_0072088 in ESCC tissues and cells. (a) The top ten circRNAs with elevated expression were validated in 25 cases of ESCC and adjacent tissues by qRT-PCR. (b) circ_0072088 expression was determined in another 58 pairs of ESCC tissue and adjacent tissue by qRT-PCR. (c) circ_0072088 expression in ESCC cell lines. (d) The expression of linear ZFR was determined in 58 pairs of ESCC tissue and adjacent tissue by qRT-PCR. (e) The expression of linear ZFR in ESCC cell lines. (f and g) The expression of ZFR and circ_0072088 in TE13 cells after si-ZFR transfection was determined by qRT-PCR. ^*∗*^*P* < 0.05, ^*∗∗*^*P* < 0.01, and ^*∗∗∗*^*P* < 0.001.

Based on the previous circRNA microarray analysis of ESCC and matched adjacent tissues (*N* = 10), the top ten circRNAs with elevated expression were validated by qRT-PCR in another 25 cases of ESCC cancer tissues and matched adjacent tissues, which revealed circ_0072088 as the most significantly differentially expressed circRNAs (Figure 1(a)). Afterward, qRT-PCR was adopted to determine the expression of circ_0072088 in cancer tissues and paired adjacent tissues of another 58 ESCC patients, which confirmed the significantly increased circ_0072088 expression in ESCC tissue than in adjacent tissue (Figure 1(b)). We further analyzed the correlation of circ_0072088 expression with clinicopathological characteristics, demonstrating that the expression of circ_0072088 was significantly correlated with tumor size, invasion depth, TNM stage, and LNM (Table 1). qRT-PCR also revealed the significantly elevated circ_0072088 expression in different ESCC cell lines compared to the normal esophageal epithelial cells HET-1A (Figure 1(c)). Meanwhile, linear ZFR expression was significantly elevated in ESCC tissues and cells compared to relative controls (Figures 1(d) and 1(e)). Afterward, three si-ZFRs were designed and transfected into TE-13 cells. As a result, si-ZFR not only successfully knocked down the expression of linear ZFR but also reduced circ_0072088 expression in ESCC cells (Figures 1(f) and 1(g)). These data suggested that the overexpression of circ_0072088 may associate with the malignant phenotypes of ESCC.

### 3.2. Biological Characteristics of circ_0072088

**Figure 2:**
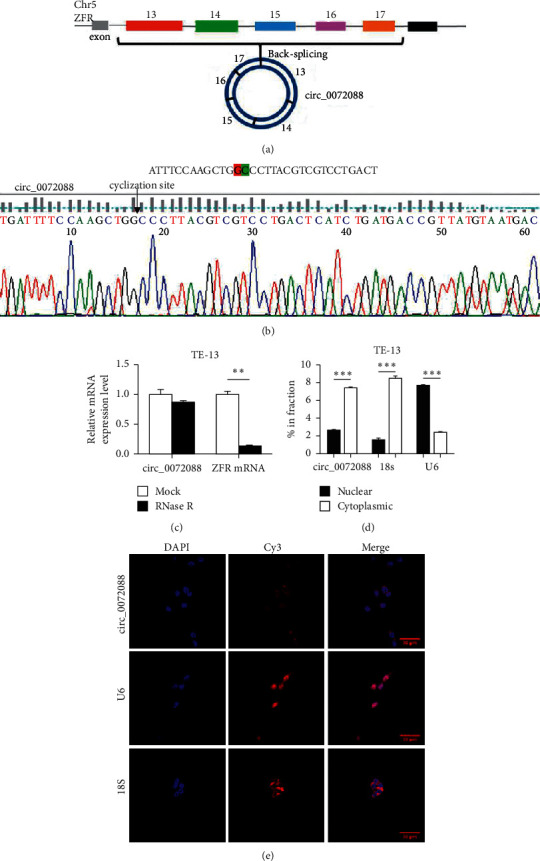
Biological characteristics of circ_0072088 in ESCC cells. (a) The origin, composition, and length of circ_0072088. (b) Sanger sequencing of circ_0072088. The black arrow indicated the reverse splice site. (c) qRT-PCR was employed to examine the expression of circ_0072088 and linear ZFR mRNA in TE-13 cells before and after RNase R treatment. (d) Nuclear and cytoplasmic separation assay of RNA was performed to understand the distribution of circ_0072088 in TE-13 cells; 18S rRNA and U6 were taken as the positive controls for cytoplasmic and nuclear components, respectively. (e) FISH assay was performed to understand the distribution of circ_0072088 in TE-13 cells; 18S rRNA and U6 were taken as the positive controls for cytoplasmic and nuclear components, respectively (Scale bars: 20 *µ*m). ^*∗∗*^*P* < 0.01, ^*∗∗∗*^*P* < 0.001.

circ_0072088 is located on chromosome 5 and is circularized by exons 13–17 on the linear ZFR mRNA, with 693 bases in length (Figure 2(a)). The circularization sites in circ_0072088 were confirmed by gene amplification and base sequencing using back-to-back primers (Figure 2(b)). RNase R treatment in the total RNA extracted from TE-13 cells and subsequent qRT-PCR demonstrated that the mRNA expression of linear ZFR was reduced after treatment, while the expression of circ_0072088 was not significantly declined, confirming that circ_0072088 was steadier than linear ZFR mRNA (Figure 2(c)). Nuclear and cytoplasmic separation and FISH assays showed that circ_0072088 was mostly located in the cytoplasm of ESCC cells (Figures 2(d) and 2(e)).

### 3.3. circ_0072088 Promotes the Proliferation, Migration, and Invasion of ESCC Cells *In Vitro*

**Figure 3:**
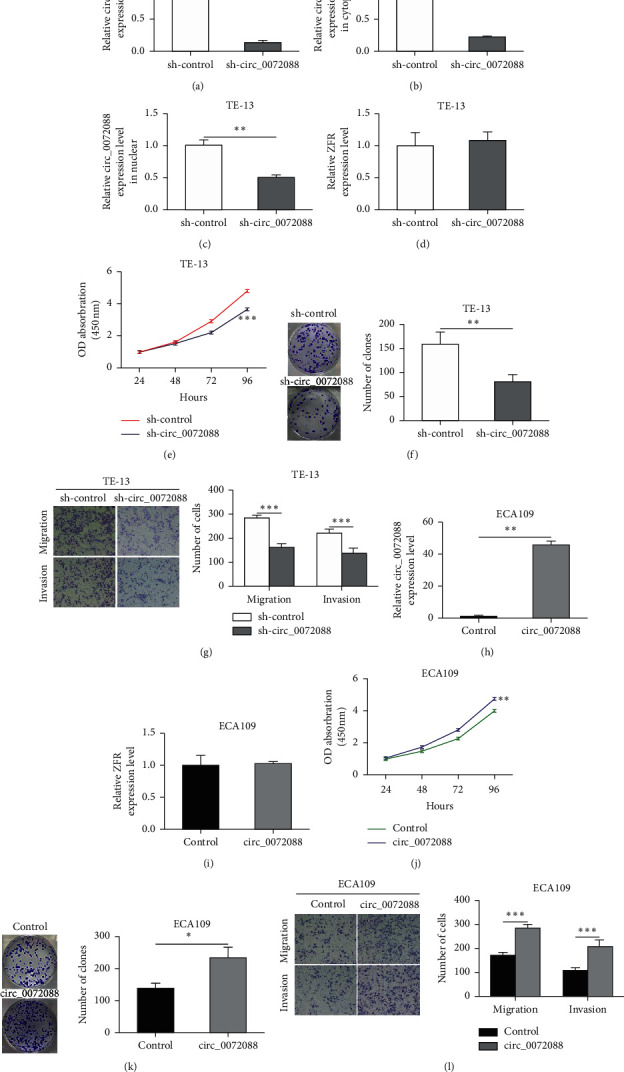
circ_0072088 enhances proliferation, migration, and invasion of ESCC cells *in vitro*. (a) qRT-PCR was utilized to determine circ_0072088 expression in TE-13 cells after sh-circ_0072088 transfection. (b and c) Nuclear and cytoplasmic separation assay for RNA was conducted for examining the expression alterations of circ_0072088 in the nucleus and cytoplasm in TE-13 cells after sh-circ_0072088 transfection. (d) qRT-PCR was used to detect the expression level of linear ZFR in TE-13 cells after sh-circ_0072088 transfection. (e) CCK-8 assay was employed for assessing TE-13 cell proliferation at 24 h, 48 h, 72 h, and 96 h after circ_0072088 downregulation. (f) The proliferation of TE-13 cells after circ_0072088 downregulation was assessed by cell colony formation. (g) Transwell assay was employed for examining migration and invasion changes in TE-13 cells after the downregulation of circ_0072088. (h and i) qRT-PCR was utilized to measure the expression of circ_0072088 and linear ZFR in ECA109 after transfection with circ_0072088 overexpression plasmid. (j) The proliferation of ECA109 cells at 24 h, 48 h, 72 h, and 96 h after the overexpression of circ_0072088 was measured by CCK-8 assay. (k) Cell colony formation assay was utilized for examining ECA109 cell proliferation after circ_0072088 overexpression. (l) Transwell assay was adopted for assessing the roles of circ_0072088 overexpression on migration as well as invasion of ECA109 cells. ^*∗*^*P* < 0.05, ^*∗∗*^*P* < 0.01, and ^*∗∗∗*^*P* < 0.001.

To further examine the roles of circ_0072088 on ESCC cells, lentiviral sh-circ_0072088 was designed and transfected into TE-13 cells. qRT-PCR further showed that sh-circ_0072088 transfection could significantly decrease circ_0072088 expression in TE-13 cells (Figure 3(a)), which was more obvious in the cytoplasm than in the nucleus (Figures 3(b) and 3(c)). However, knocking down circ_0072088 did not affect the expression of linear ZFR (Figure 3(d)). CCK-8 and colony formation assays showed that the proliferation capacity of TE-13 cells was significantly decreased after knocking down circ_0072088 (Figures 3(e) and 3(f)). Transwell assay revealed that the migration and invasion abilities of TE-13 cells were significantly decreased after the downregulation of circ_0072088 (Figure 3(g)).

The biological effects of circ_0072088 on malignant phenotypes of ESCC were also verified in ECA109 cells. Transfection of circ_0072088 overexpression plasmid into ECA109 cells could significantly increase the expression level of circ_0072088, which did not affect the expression of linear ZFR (Figures 3(h) and 3(i)). In addition, circ_0072088 overexpression could enhance the proliferation, migration, and invasion of ESCC cells (Figures 3(j)–3(l)).

### 3.4. circ_0072088 Acts as a Sponge for miRNA-377

**Figure 4:**
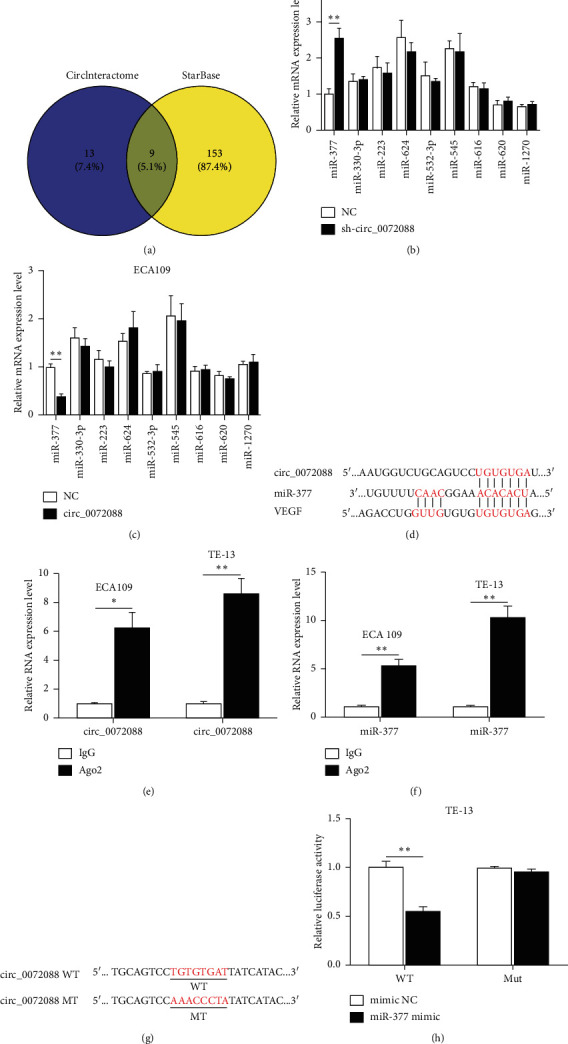
circ_0072088 acts as a sponge for miRNA-377. (a) Venn diagram showed nine common downstream target genes of circ_0072088 predicted by two databases (CircInteractome and starBase). (b) Expression alterations in downstream target genes after the downregulation of circ_0072088 in TE-13 cells. (c) Expression alterations in downstream target genes after the overexpression of circ_0072088 in ECA109 cells. (d) Schematic diagram of the binding site of circ_0072088-miRNA-377-VEGF. (e) RIP assays confirmed the binding of circ_0072088 and Ago2 protein in ECA109 and TE-13 cells. (f) RIP assays confirmed the binding of miRNA-377 and Ago2 protein in ECA109 and TE-13 cells. (g) The mutation site of dual-luciferase reporter plasmid of circ_0072088. (h) Dual-luciferase reporter assay was performed for the assessment of luciferase activity after cotransfection of circ_0072088-wt/circ_0072088-mut and miRNA-377 mimics. ^*∗*^*P* < 0.05, ^*∗∗*^*P* < 0.01.

Because circ_0072088 was an exon circRNA mainly located in the cytoplasm, it may act as the sponge to absorb miRNAs to regulate its downstream target genes, thereby influencing phenotypes of ESCC cells. By searching and analyzing the circular RNA interactome and starBase databases, we found that nine miRNAs had the binding sites for circ_0072088, including miRNA-223, miRNA-330-3p, miRNA-377, miRNA-532-3p, miRNA-545, miRNA-616, miRNA-1270, miRNA-620, and miRNA-624 (Figure 4(a)). The qRT-PCR assay showed that miRNA-377 expression was significantly upregulated after knocking down circ_0072088 in TE-13 cells, while the expression of other miRNAs was not significantly changed (Figure 4(b)). Similar data were received from ECA109 cells. After the upregulation of circ_0072088 in ECA109 cells, the expression of miRNA-377 was significantly downregulated, while the expression of other miRNAs was not significantly changed (Figure 4(c)). These results suggested that circ_0072088 could regulate the expression of miRNA-377. The binding site of miRNA-377 to circ_0072088 was then predicted by TargetScan (Figure 4(d)). RIP assay was performed to examine whether miR-377 could bind to circ_0072088 by means of Argonaute protein. The result indicated that the expression of circ_0072088 and miRNA-377 was significantly increased in the Ago2 group than in the IgG group in ECA109 and TE-13 cells (Figures 4(e) and 4(f)). Afterward, circ_0072088-wt and circ_0072088-mut dual-luciferase reporter plasmids were designed and synthesized according to the binding sites between circ_0072088 and miRNA-377 (Figure 4(g)). circ_0072088-wt/circ_0072088-mut plasmid and miRNA-377 mimic or miRNA-NC mimic were cotransfected into TE-13 cells, revealing that miRNA-377 could only decrease the luciferase activity of the circ_0072088-wt plasmid, but not circ_0072088-mut plasmid (Figure 4(h)). These results confirmed that circ_0072088 could act as a sponge to absorb miRNA-377 in ESCC cells.

### 3.5. circ_0072088 Acts as a Sponge for miRNA-377 to Target VEGF

**Figure 5:**
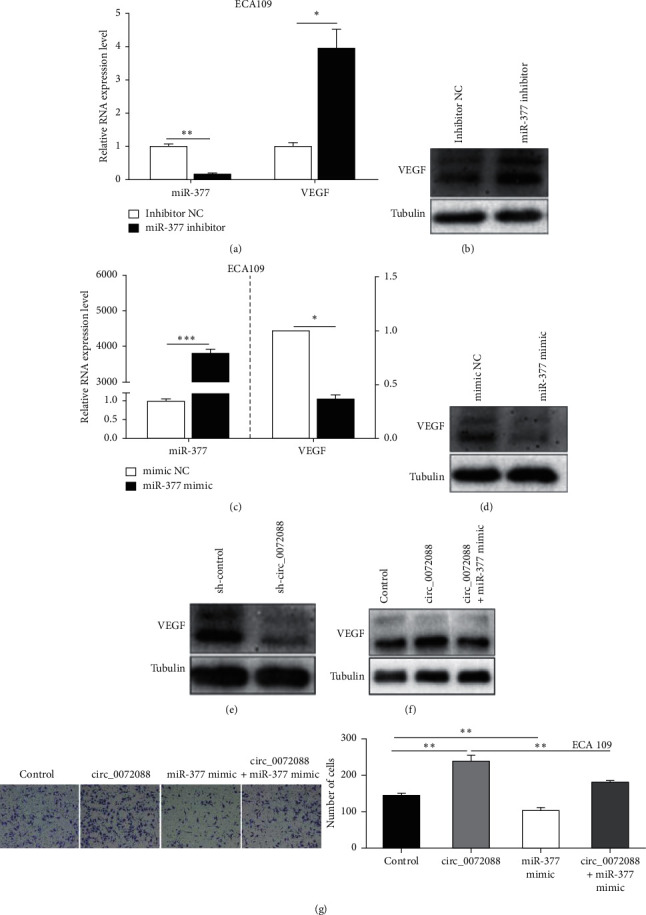
circ_0072088 acts as a sponge for miRNA-377 to target VEGF to further promote the migration of ESCC cells. (a–d) qRT-PCR and Western blot were employed for examining the effects of downregulation and upregulation of miRNA-377 on the VEGF gene and protein expression. (e) The regulatory role of circ_0072088 downexpression on the VEGF protein level was examined by Western blot. (f) Western blot was adopted for determining the regulatory effect of circ_0072088 overexpression on the VEGF protein level and the regulatory role of miRNA-377 mimic on the upregulation of VEGF induced by circ_0072088 overexpression. (g) Transwell assay was conducted for examining the functions of miRNA-377 overexpression on the migration ability of ECA109 cells with circ_0072088 overexpression. ^*∗*^*P* < 0.05, ^*∗∗*^*P* < 0.01, and ^*∗∗∗*^*P* < 0.001.

The prediction data showed that VEGF is one of the potential binding targets of miRNA-377. Western blot and qRT-PCR demonstrated that the downregulation of miRNA-377 in ECA109 cells could significantly increase both mRNA and protein expression levels of VEGF (Figures 5(a) and 5(b)), while the upregulation of miRNA-377 in ECA109 cells could significantly inhibit both mRNA and protein expression levels of VEGF (Figures 5(c) and 5(d)), indicating that VEGF was a direct target gene of miRNA-377. Western blot showed that circ_0072088 downregulation could significantly decrease the protein level of VEGF (Figure 5(e)), while circ_0072088 upregulation could significantly increase the protein level of VEGF (Figure 5(f)). Meanwhile, Western blot revealed that the overexpression of miRNA-377 could reverse the upregulatory effects of circ_0072088 on VEGF protein (Figure 5(f)). Transwell assay showed that the overexpression of miRNA-377 could reverse the promoting effects of circ_0072088 on migration in ECA109 cells (Figure 5(g)). The above findings indicated that circ_0072088 could promote the migration of ESCC cells through upregulating VEGF by acting as a sponge for miRNA-377.

### 3.6. circ_0072088 Promotes ESCC Cell Proliferation *In Vivo*

**Figure 6:**
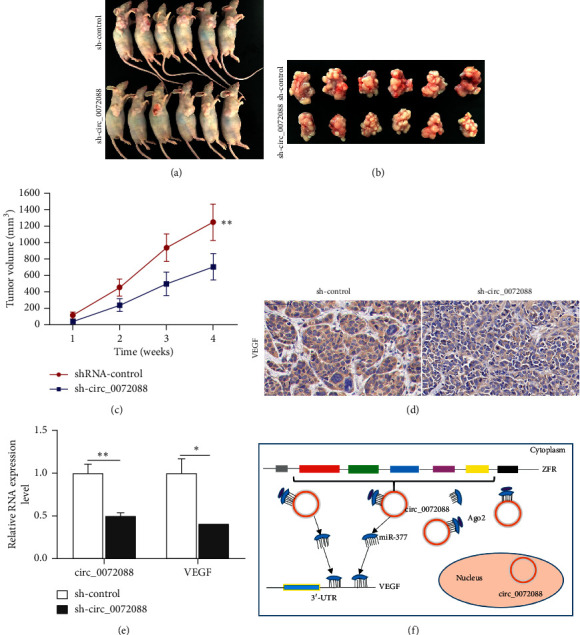
circ_0072088 promotes ESCC cell proliferation *in vivo*. (a–c) Tumor size of mice after subcutaneous injection of TE-13 cells in the right axilla for 28 days. (d) IHC for tumor tissue of mice from two groups (scale bars: 50 *µ*m). (e) The expression alteration of circ_0072088 and VEGF in tumor tissue of mice from two groups was assessed by qRT-PCR. (f) Schematic diagram of the circ_0072088-miR-377-VEGF regulatory signal axis. ^*∗*^*P* < 0.05, ^*∗∗*^*P* < 0.01.

To confirm the functions of circ_0072088 on ESCC cell proliferation *in vivo*, sh-circ_0072088 TE-13 cells and sh-control TE-13 cells were subcutaneously injected into the right axilla of 6-week-old nude mice, respectively (4 × 10^6^/each mouse). Tumor volume was determined every week, and mice were sacrificed 28 days after subcutaneous injection. As a result, tumor growth was significantly lower in the sh-circ_0072088 group than in the control group. After 28 days, tumor volume was significantly smaller in the sh-circ_0072088 group than in the control group (Figures 6(a)–6(c)). IHC showed significantly lower VEGF expression of tumor tissue in the sh-circ_0072088 group than in the sh-control group (Figure 6(d)). qRT-PCR indicated that VEGF expression in tumor tissue was significantly lower in the sh-circ_0072088 group than in the sh-control group (Figure 6(e)).

## 4. Discussion

circRNA, as a novel endogenous RNA, has recently become a research focus. Accumulative studies have shown that circRNAs not only play roles in carcinogenesis and tumor progression but also might be novel biomarkers for early diagnosis as well as therapeutic targets of tumors [15].

The expression profiles of our previous circRNA chip from 10 pairs of ESCC and matched adjacent tissues were validated, which further identified circ_0072088 as the most significantly differentially expressed circRNA. In this study, we further confirmed that the expressions of circ_0072088 and linear ZFR were increased in ESCC tissues and cells. si-ZFR transfection in ESCC cells could decrease linear ZFR expression and simultaneously reduce circ_0072088 expression, indicating that the elevated expression circ_0072088 in ESCC tissues and cells was partially due to the increased expression of linear ZFR. In addition, the expression of circ_0072088 in ESCC was positively related to tumor size, invasion depth, clinical stage, and LNM. Large tumor size, deep invasion, advanced clinical stage, and LNM in ESCC patients suggest poor prognosis [16–19]; therefore, circ_0072088 might be adopted as a diagnostic and prognostic predictor of ESCC.

circRNAs are enriched in circulating exosomes, plasma, and cells, which are more stable than linear RNAs [15, 20]. In this study, RNase R treatment was performed, which confirmed that circ_0072088 was steadier than linear ZFR mRNA. However, it requires more in-depth investigations on whether the plasma level of circ_0072088 could be used as a diagnostic and prognostic biomarker for ESCC.

Because high expression of circ_0072088 was related to the aggressive phenotype of ESCC, we hypothesized that circ_0072088 was likely to be involved in tumor biology. To begin with, *in vitro* functional assays validated that high expression of circ_0072088 promoted the proliferation, migration, and invasion of ESCC cells, which was inhibited by circ_0072088 downregulation. Consistently, circ_0072088 downregulation could suppress ESCC cell proliferation *in vivo*.

ZFR is located at 5p13.3 and with 20 exons and 97416 in length, which encodes a zinc finger protein. circ_0072088 is circularized from linear ZFR and mainly exists in the cytoplasm as an exon circRNA [21]. In addition, nuclear and cytoplasmic separation assay and FISH assay also confirmed that circ_0072088 was mainly located in the cytoplasm, indicating that it was able to posttranscriptionally regulate miRNAs.

The functions of circRNAs include miRNA sponges, regulation of alternative splicing and gene transcription, and protein decoys [5, 22]. Among them, the sponge absorption function of circRNA has been most studied. circRNAs can function as the miRNA sponges to competitively absorb miRNAs to attenuate the inhibitory effects of miRNAs on their target genes, thereby affecting tumorigenesis and tumor progression [23–25]. By analyzing and predicting circ_0072088-related miRNAs, miRNA-377 was screened by interfering with the expression level of circ_0072088 in ESCC cells and miR-377 contains seven paired nucleotides with circ_0072088. MiRNAs can form the RNA-induced silencing complex (RISC) with Argonaute protein to silence or degrade the target mRNA through base pairing [26]. In this study, the RIP experiment indicated that the relative enrichment of circ_0072088 and miR-377 in the Ago2 coprecipitation group was remarkably increased relative to that in the IgG immunoprecipitation group, followed by dual-luciferase reporter assay to confirm the direct interaction between circ_0072088 and miRNA-377.

miRNA-377 has been revealed as a tumor suppressor gene to regulate various phenotypes in tumors [27, 28]. The expression of miR-377 is significantly lower in the ESCC tissue than in the adjacent tissue, and the miR-377 expression level is negatively related to the clinical stage as well as distant metastasis but is positively related to the survival rate of patients [29].

The growth and metastasis of invasive tumors are closely associated with angiogenesis [30]. VEGF, the most important regulatory factor in angiogenesis, is a mitogenic factor to promote the proliferation of endothelial cells and angiogenesis, which is continuously overexpressed throughout the tumor life cycle [31, 32]. VEGF is widely expressed in multiple types of malignancies, including lung cancer, EC, head and neck squamous cell carcinoma, colorectal cancer, breast cancer, and ovarian cancer, and the high expression of VEGF is positively related to LNM and tumor angiogenesis [29, 31–34]. VEGF can be regulated by a variety of miRNAs. For example, miR-655 inhibits the proliferation and invasion of ovarian cancer cells by directly targeting VEGF [35]. Low expression of miR-125a inhibits the proliferation and metastasis of hepatocellular carcinoma cells by targeting VEGF [36]. Li et al. have reported that miR-377 can bind to the 3′-UTR of VEGF and play a negative regulatory role in EC [31].

In this study, we have also confirmed that miR-377 negatively regulates VEGF in ESCC by Western blot and simultaneously validated the mechanism of the regulatory role of circ_0072088 on VEGF expression in ESCC. In other words, circ_0072088 can act as a sponge for miR-377 to attenuate the inhibitory effect of miR-377 on VEGF expression, which could elevate the expression of VEGF, thereby promoting proliferation, migration, and invasion of ESCC. These findings sufficiently illustrate that the circ_0072088-miR-377-VEGF signaling axis is vitally involved in regulating ESCC (Figure 6(f)).

## 5. Conclusion

We demonstrated the elevated expression of circ_0072088 in ESCC tissues and cells, which was positively related to tumor size, invasion depth, clinical stage, and LNM in ESCC patients. Functionally, circ_0072088 could enhance proliferation, migration, and invasion of ESCC cells *in vitro*, and circ_0072088 downregulation was capable of suppressing ESCC cell proliferation *in vivo*. circ_0072088 could regulate the expression of VEGF to promote proliferation, migration, and invasion of ESCC by acting as a sponge for miR-377. Collectively, circ_0072088 might be used as a new molecular marker for early diagnosis and therapeutic target for ESCC.

^*∗*^ *P* < 0.05, ^*∗∗*^*P* < 0.01.

## Figures and Tables

**Table 1:** Correlation between circ_0072088 expression level and clinicopathological features in ESCC patients.

Characteristics	All patients	circ_0072088 low expression	circ_0072088 high expression	*χ* ^2^	*P* values
*Age (years)*				1.673	0.196
<60	6	5	1		
≥60	52	24	28		

*Gender*				2.479	0.115
Male	45	25	20		
Female	13	4	9		

*Tumor size (cm)*				4.678	0.030^*∗*^
<5 cm	36	22	14		
≥5 cm	22	7	15		

*Histological grade*				3.395	0.065
Moderately	31	19	12		
Poorly	27	10	17		

*Tumor infiltration depth*				5.695	0.017^*∗*^
T1-T2	33	21	12		
T3-T4	25	8	17		

*TMN stage*				8.345	0.004^*∗∗*^
I-II	29	20	9		
III-IV	29	9	20		

*Lymph metastasis*				8.345	0.004^*∗∗*^
Yes	29	9	20		
No	29	20	9		

## Data Availability

All data and materials are available upon request from the corresponding author.

## Supplementary Materials

Supplementary Materials Supplementary Table 1 (Table S1): the primers and their sequences for PCR (5′-3′). Supplementary Table 2 (Table S2): the sh-circ_0072088, si-ZFR, miR-377 mimic/NC, and miR-377 inhibitor/NC sequences used in experiments.
