# Supplementary material for: Circ_0072088 Promotes Proliferation, Migration, and Invasion of Esophageal Squamous Cell Cancer by Absorbing miR-377
Source: J Oncol. 2020 Sep 29;2020:8967126. doi: 10.1155/2020/8967126 (PMC7542490; doi:10.1155/2020/8967126)
Supplement: Supplementary Materials — Supplementary Table 1 (Table S1): the primers and their sequences for PCR (5′-3′). Supplementary Table 2 (Table S2): the sh-circ_0072088, si-ZFR, miR-377 mimic/NC, and miR-377 inhibitor/NC sequences used in experiments. [file 8967126.f1.doc]

**Supplementary Table 1 The primers and their sequences for PCR (5’-3’)**

| **Primer name** | **Sequence** **5’****-3’** |
| --- | --- |
| circ_0072088 For | TGATTTTCCAAGCTGGCCCT |
| circ_0072088 Res | TCTGAACTGCCTGTAACTCC |
| VEGF For | TGCCCACTGAGGAGTCCAAC |
| VEGF Res | TGGTTCCCGAAACGCTGAG |
| 18S For | GGAGTATGGTTGCAAAGCTGA |
| 18S Res | TCCTGCTTTGGGGTTCGATT |
| U6 For | CTCGCTTCGGCAGCACA |
| U6 Res | AACGCTTCACGAATTTGCGT |
| GAPDH For | GGGAAGCTCACTGGCATGGCCTTCC |
| GAPDH Res  ZFR For | CATGTGGGCCATGAGGTCCACCAC  TGTGCTGTATCTGAAGCGGC |
| ZFR Res  miR-223 For  miR-223 Res  miR-330-3p For  miR-330-3p Res  miR-377 For  miR-377 Res  miR-624 For  miR-624 Res  miR-532-3p For  miR-532-3p Res  miR-545 For  miR-545 Res  miR-616 For  miR-616 Res  miR-1270 For  miR-1270 Res  miR-620 For  miR-620 Res | CCGGTGGGTCTTTCACCATA  GCCCGCCAGUUUGUCAAAUA  GTGCAGGGTCCGAGGT  GCAGAGATTCCGTTGTCGT  GCGAGCACAGAATTAATACGAC  GCACCAATCACACAAAGGCA  TATGGTTGTTCACGACTCCTTCAC  AAGACAAAATTTATTTTCCAGGGATTTAATACGTAC  GTGAAAAGATGTTTTGTCACAGGAAAAAGGAAATCC  ACACTCCCCTCCCACACCCAAGG  CTCAACTGGTGTCGTGGAGTCGGCAATTCAGTTGAG  TCAGTAAATGTTTATTAGATGA  GTGCAGGGTCCGAGGTATTC  CCTGTACGCCAACACAGTGC  ATACTCCTGCTTGCTGATCC  CTGGAGATATGGAAGAGCTGTGT  TGCAAAGAGCCACATAGAAGAT  ACACTCCAGCTGGGATGGAGATAGATAT  CTCAACTGGTGTCGTGGAGTCGGCAATTCAGTTGAGATTTCTAT |

**Supplementary Table 2 The sh-circ_0072088, si-ZFR, miR-377 mimic/NC and miR-377 inhibitor/NC sequences used in experiments**

| **Name** | **Sequence (5’-3’)** |
| --- | --- |
| sh-circ_0072088  si-ZFR#1 sense  si-ZFR#1 antisense  si-ZFR#2 sense  si-ZFR#2 antisense  si-ZFR#3 sense  si-ZFR#3 antisense  miR-377 mimic sense  miR-377 mimic antisense  miR-377 mimic-NC sense  miR-377 mimic-NC antisense  miR-377 inhibitor  miR-377 inhibitor-NC | TTTCCAAGCTGGCCCTTACGT  CCAGGAUUCAUACUCAUAUTT  AUAUGAGUAUGAAUCCUGGTT  GGUUACAGCCAAGGUGCAATT  UUGCACCUUGGCUGUAACCTT  GGUAAUAAGCUGCAGUCAATT  UUGACUGCAGCUUAUUACCTT  AUCACACAAAGGCAACUUUUGU  AAAAGUUGCCUUUGUGUGAUUU  UUCUCCGAACGUGUCACGUTT  ACGUGACACGUUCGGAGAATT  ACAAAAGUUGCCUUUGUGUGAU  CAGUACUUUUGUGUAGUACAA |
